# Multiple uses of fibrin sealant for nervous system treatment following injury and disease

**DOI:** 10.1186/s40409-017-0103-1

**Published:** 2017-03-14

**Authors:** Natalia Perussi Biscola, Luciana Politti Cartarozzi, Suzana Ulian-Benitez, Roberta Barbizan, Mateus Vidigal Castro, Aline Barroso Spejo, Rui Seabra Ferreira, Benedito Barraviera, Alexandre Leite Rodrigues Oliveira

**Affiliations:** 1 Graduate Program in Tropical Diseases, Botucatu Medical School, Univ Estadual Paulista (UNESP), Botucatu, SP Brazil; 2 Center for the Study of Venoms and Venomous Animals (CEVAP), Univ Estadual Paulista (UNESP), Botucatu, SP Brazil; 3 Department of Structural and Functional Biology, Institute of Biology, University of Campinas (UNICAMP), Laboratory of Nerve Regeneration, CEP 13083-970 Campinas, SP Brazil; 4 Neuro Development Lab, School of Biosciences, University of Birmingham, Birmingham, England UK; 5 The School of Medicine at Mucuri (FAMMUC), Federal University of Jequitinhonha and Mucuri Valleys (UFVJM), 39803-371 Teófilo Otoni, MG Brazil

**Keywords:** Central nervous system, Peripheral nervous system, Commercial fibrin sealant, New heterologous fibrin sealant, Nervous system injury, Fibrin tissue adhesive

## Abstract

Lesions to the nervous system often produce hemorrhage and tissue loss that are difficult, if not impossible, to repair. Therefore, scar formation, inflammation and cavitation take place, expanding the lesion epicenter. This significantly worsens the patient conditions and impairment, increasing neuronal loss and glial reaction, which in turn further decreases the chances of a positive outcome. The possibility of using hemostatic substances that also function as a scaffold, such as the fibrin sealant, reduces surgical time and improve postoperative recovery. To date, several studies have demonstrated that human blood derived fibrin sealant produces positive effects in different interventions, becoming an efficient alternative to suturing. To provide an alternative to homologous fibrin sealants, the Center for the Study of Venoms and Venomous Animals (CEVAP, Brazil) has proposed a new bioproduct composed of certified animal components, including a thrombin-like enzyme obtained from snake venom and bubaline fibrinogen. Thus, the present review brings up to date literature assessment on the use of fibrin sealant for nervous system repair and positions the new heterologous bioproduct from CEVAP as an alternative to the commercial counterparts. In this way, clinical and pre-clinical data are discussed in different topics, ranging from central nervous system to peripheral nervous system applications, specifying positive results as well as future enhancements that are necessary for improving the use of fibrin sealant therapy.

**Electronic supplementary material:**

The online version of this article (doi:10.1186/s40409-017-0103-1) contains supplementary material, which is available to authorized users.

## Background

The nervous system is immensely complex and responsible for most of the biological responses and maintenance of homeostasis. It is, however, subject to injuries and pathologies that usually require surgical intervention. Due to its cellular organization, high vascularization and the presence of the blood–brain barrier, to interfere in the nervous tissue parenchyma constitutes a major challenge. The possibility of using a biological scaffold to provide hemostasis, reestablishment of subarachnoid space tightness as well as a vehicle for drug and stem cell delivery opened a new and promising field of research.

The use of homologous commercial fibrin sealants (FS) in a number of surgical procedures is now consolidated as an efficient method to avoid suturing, enhancing success ratio and reducing patient recovery time. To provide an alternative to human blood derived fibrin sealants, the Center for the Study of Venoms and Venomous Animals (CEVAP – UNESP) has proposed a new heterologous bioproduct composed of certified animal components, including a thrombin-like enzyme obtained from snake venom and a buffalo-cryoprecipitate rich in fibrinogen [1–4]. After more than 20 years of efforts, this study is now under clinical trials [5–10].

The present review positions CEVAP heterologous fibrin sealant (HFS) in the context of nervous system repair following trauma and diseases, indicating a possible advantageous use in different instances. Recent literature is provided and discussed in different topics, ranging from central nervous system (CNS) to peripheral nervous system (PNS) applications, specifying positive results as well as future enhancements that are necessary for improving the use of fibrin sealant therapy.

## Brain

The use of FS in brain lesions is not restricted to its common use in the treatment and prevention of cerebrospinal fluid (CSF) leaks [11]. It has expanded to a wide range of surgical interventions including as a hemostatic agent following the total or partial extraction of brain tumors and for coaptation of nerves and brain vessels, replacing conventional sutures [11–13]. There are also promising results from combining this valuable adjunct with various drugs and other agents to enhance regenerative and therapeutic effects after a wide spectrum of brain traumas whether accidental, surgical or even congenital [14–17]. Since the early 20^th^ century, fibrin has been used for stopping cerebral hemorrhage and it is currently being employed through the sealant in various reparative procedures [2, 18].

A cerebrospinal fluid fistula is a condition in which there is a leak of CSF to the nasal cavity, due to fracture of the skull, resulting from traumatic causes (accidental or surgical) and non-traumatic causes, also known as spontaneous fistulas [11, 19]. In both cases, the persistent leakage of CSF might cause complications that are responsible for significant mortality and morbidity [20]. Most leaks provoked by head trauma will seal without intervention; however, spontaneous or surgically-induced leaks often require operative repair [20].

Some authors described treatment by FS of acute (intraoperative) cerebrospinal fluid leaks [21, 22]. Green et al. [21] evaluated FS as an adjunct to sutured dural repair to obtain intraoperative watertight closure in patients undergoing elective cranial surgery. The study demonstrated the superiority of FS over sutures in establishing intraoperative tight closure of a dural incision. Furthermore, Hobbs et al. [22] demonstrated the effectiveness of FS in 120 patients undergoing pituitary surgery procedures with intraoperative CSF leaks. All intraoperative leaks were managed using the FS with different materials, resulting in a low incidence of postoperative CSF leakage.

Other authors described FS as preventing postoperative cerebrospinal fluid leaks [23, 24]. Its use was predominantly in cranial procedures with low incidences of postoperative CSF leaks [11]. Many cases involving patients undergoing transsphenoidal surgery in which postoperative CSF leaks significantly decreased were reported [11]. For example, Yoshimoto et al. [23] evaluated a FS for prevention of postoperative extra dural fluid collection through the dural sutures in patients undergoing craniotomy for an unruptured aneurysm. Once again, the study demonstrated the superiority of the fibrin sealant over sutures. Furthermore, a retrospective (historical) study by Kassam et al. [24] evaluated the efficacy and cost-effectiveness of fibrin in patients with intracranial pathological lesions. The incidence of CSF leaking in matched groups treated with FS or without it were compared. There were no cases of CSF leak in the group of patients receiving FS. Thus, the authors conclude that the FS reduces the incidence of postoperative CSF leaks.

Recent studies in animal models are corroborating the hypothesis that FS prevents CSF leakage. Hutchinson et al. [25] compared two available FS with a synthetic polyethylene glycol (PEG) hydrogel sealant in a canine durotomy repair model. This well-characterized model employed 27 mongrel dogs to assess the ability of sealants to achieve intraoperative tight seals of the dura mater, as well as long-term safety and efficacy. The application of these sealants was 100% effective in preventing CSF leakage.

Finally, a few authors described FS as a treatment for persistent CSF leaks. Cappabianca et al. [26] locally injected FS in patients following different neurosurgical procedures. The injection of FS has proven to be effective in filling or sealing postoperative recesses and treating minor or initial CSF leaks, adding another possibility for threatening postoperative leaks.

Besides CSF leaks, postoperative subdural fluid collection (SFC) is another complication of craniotomy, being most frequently employed after aneurysm surgery [27]. Most SFC cases eventually disappear or are clinically asymptomatic. However, some SFCs enlarge, leading to hygromas or subdural hematomas, which require surgical treatment [27]. In this sense, arachnoid plasty has been demonstrated to be effective for preventing SFC. Several arachnoid plasty methods have been reported including its sealing with FS or covering with appropriate materials and FS. Thus, Abe et al. [27] examined the efficacy of arachnoid plasty with collagen sheet and FS after the clipping of unruptured aneurysms. The procedure achieved favorable outcomes with zero incidence of SFC or complications such as surgical infection.

Lee et al. [12] described a series of 26 patients who underwent microneurosurgical operations in which FS was used. The patients had various neurological disorders: 11 had cerebral aneurysms, 11 had brain tumors, two had lipomyelomeningoceles, one had cerebral arteriovenous malformation and one had torn dura resulting from a mastoidectomy. The FS was tested and effective in the following procedures: reinforcement of aneurysmal clipping; local hemostasis; protection of cerebral veins and sealing of CSF leakage.

Fujimura et al. [13] studied the incidence of chronic hydrocephalus by analyzing a series of 39 patients with subarachnoid hemorrhage, who underwent perivascular coating with FS of cerebral arteries after clipping of an aneurysm. The authors concluded that there were no complications caused by FS and that it protected the cerebral arteries during the acute phase.

Furthermore, there are also promising results associating fibrin sealants with other components, even in cell therapy. An example of this association is the combination of collagen foil or fleece with FS. It is known that the collagen has been successfully employed as a dural graft for years, but when used in combination with fibrin sealant, it enhances sealing and tissue regeneration properties, positively reflecting on hemostasis and stimulation of tissue repair. Besides, such combination prevents fibrin sealant to be washed away in cases of CSF leakage. Thus, a combination of collagen and FS is effective, safe and biocompatible. No further adverse events, complications or toxicity were reported [14–16, 28, 29].

Another example is the FS association with stem cells. Chen et al. [17] investigated the therapeutic effects of subdural transplantation of inducible pluripotent stem cells (iPS) mixed with fibrin sealant (iPS-FS) on rats with cerebral ischemia induced by middle cerebral artery occlusion (MCAO). They demonstrated that subdural iPS-FS enhances recovery from induced stroke and is able to avoid iatrogenic injury to brain parenchyma, thus comprising a safer alternative approach. In this respect, due to the feasibility of obtaining formulations with varying characteristics (customization), the use of the derived snake venom sealant enables an association with potentially different compounds beneficial for the regeneration process of the nervous system.

Thus, it is evident that the FS is a valuable adjuvant to various microneurosurgical procedures, and potentially useful by contributing to the improvement of surgical techniques related to different disorders and adversities in the brain and surrounding environment.

## Spinal cord

Spinal cord injury (SCI) by compression or spondylolisthesis usually results in cavitation and glial scar formation. Biomatrices with immunomodulatory properties are of interest since they may be used to bridge the lesion, reducing the formation of scar tissue, as well as facilitating axonal growth. In this context, FS could act as a carrier for therapeutic agents, such as neurotrophic factors and stem cells [30–32].

Guest et al. [33] combined fibroblast growth factor (FGF) and FS to human Schwann-cell grafts which were engrafted to transected rat spinal cords. Such therapy reduced retrograde axonal degeneration stimulating fiber regeneration throughout the implant. In human patients, a therapeutic combination of FGF and FS was applied to the injured spinal segment and used to prevent postoperative CSF leakage. The treatment resulted in significant motor and sensory improvements [34].

FS can be complexed with FGF and nerve grafts as well. Kuo et al. [35] used autologous peripheral intercostal nerve segments combined with FGF in an FS scaffold, implanted to bridge the 5 mm gap in transected rat spinal cords. FGF treatment induced IL-4 expression while nerve grafts induced nerve grow factor (NGF) and brain-derived neurotrophic factor (BDNF) expression. This combined treatment has also been applied to animals with chronic complete SCI by the removal of scar tissue to expose fresh tissue at the surface of the spinal cord stumps [36]. Such approach restored a degree of hind-limb function [36, 37]. Tsai et al. [38] also treated spinal cord transection with peripheral nerve grafts and spinal cord anastomosis, both including FGF1 in an FS scaffold. Rats recovered both motor-evoked potentials, recorded at the lumbar level and locomotor function due to long tract regeneration.

Proteins can be complexed with fibrin matrix. Lord-Fontaine et al. [39] used rat contusion model and topical application of the protein BA-210 onto the spinal cord using an FS formulation. BA-210 inactivates Rho, which activation is a conserved response in various types of central injuries, thus significantly reducing tissue loss in the perilesional area and rostrocaudal spreading of lesion cavity. Significant walking abilities were regained more rapidly and more consistently in rats treated with BA-210 [39]. Although a previous work has shown a potential scaffold role for FS, which enhanced FGF and BA-210 treatments, FS action itself has not been fully evaluated [39].

FS is already applied by neurosurgeons as a hemostatic agent and for the control of cerebrospinal fluid (CSF) leaks [15, 32, 40–42]. In this sense, postoperative CSF leakage is a known complication of spinal surgery. The ideal material to be used in the dural closure is still a matter of debate [43]. Prompt surgery is recommended to prevent the complications such as meningitis, CSF fistulas, and pseudocyst formation with potential nerve compression [44]. In this regard, FS has been considered effective for prevention of CSF leakage in the field of neurosurgery and spinal surgery [45]. Frequently, durotomy margin is uneven, and watertight dural closure cannot be achieved only by single sutures. In such cases, the use of a sealant is helpful [45–49].

Many authors recommend FS to reinforce the site of durotomy and have reported that the FS-treated patients presented a significantly higher rate of tight closure than controls as well as decreased postoperative drainage output and time spent at the hospital [45, 48–51]. Percutaneous therapy of FS in humans with postoperative CSF leaks generated a 50% success rate, similar to the 56% success rate in rats with direct application of FS alone, after experimental induction of CSF rhinorrhea [44, 52]. Patel et al. [44] recommend autologous cryoprecipitate use whenever possible to avoid the risk of blood-borne pathogens, including hepatitis C. The preparation of cryoprecipitate from autologous blood requires three days, and 500 mL of whole blood generates 20 to 25 mL of cryoprecipitate [44].

An autologous FS has also been used by Nakamura et al. [45] – in comparison to commercial FS – in patients undergoing spinal surgery. No complications such as infection or continuous CSF leak were observed in any case. The volume of drainage fluid was significantly reduced in the group subjected to either autologous or commercial FS, when compared to the group without FS. As to safety, the autologous adhesive was equal to the commercial counterpart. The preventive effects of both adhesives were equivalent, but the autologous adhesive is much cheaper and provides the advantage of being risk-free of transfusion infection.

As a treatment for sacral meningeal cysts, Paulsen et al. [53] determined whether placement of FS after aspiration could offer a more definitive therapy. The use of FS resulted in marked improvement in all patients, with no evidence of pathology recurrence [54].

Although FS use has produced positive results, there are reports of inconsistent outcomes. Thus, in a retrospective analysis done by Balasubramaniam et al. [43], evaluating children submitted to surgery for various spinal pathologies, FS had no effect, though the numbers were statistically too small. Jankowitz et al. [55] reached a similar conclusion that the use of FS did not significantly decrease the incidence of subsequent CSF leakage while studying the potential efficacy of FS TISSEEL® (Baxter) for enhancing dural repair after lumbar spine surgery. Considering the risk of healing inhibition, the findings did not support the prophylactic use of FS when a primary repair is deemed adequate. Augmentation with muscle, fat, FS, or graft should be considered when the dural closure is suboptimal.

When used to fill the lesion gap after SCI, FS provided neuroprotective effects. Tissucol® (Baxter) FS was used by Petter-Puchner et al. [32] after thoracic spinal cord hemisection in rats. Three and seven days after lesion, histology showed a more pronounced inflammatory response triggered by macrophages in the FS-treated group. This difference did not impair behavioral or reflex tests performed at the same time points. At day 28, recruitment of macrophages and microglia had substantially decreased and no intergroup difference was detectable. Substantial benefits were found in relation to motor function and proprioceptive recovery in the FS-treated group [32]. A similar result was achieved after intramedullary axotomy and a new heterologous fibrin sealant (HFS) treatment. The HFS-treated group displayed improved motoneuronal survival after lesion and showed upregulation of iNOS2 and arginase­1 genes, proinflammatory (TNF­α and IL­1β) and anti­inflammatory cytokines (IL­10, IL­4, and IL­13). Thus, HFS enhanced early macrophage recruitment and proinflammatory cytokine expression, which contributed to an acceleration of inflammation resolution, shown by the increased expression of M2 macrophage markers and anti­inflammatory cytokines. The greater inflammation was coupled with better motor performance in the walking track test [56].

## Spinal cord ventral and dorsal roots

Spinal motoneurons are located in the spinal cord ventral horn and send their axon towards the periphery to innervate skeletal muscles. These efferent fibers, among other functions, control the voluntary movements in response to central brain stimulation and/or sensory feedback. Afferent fibers bring sensorial information (touch, temperature, pressure, pain and proprioception) from the periphery to the CNS through the dorsal roots. Sensorial feedback and motor control are crucial in our everyday life, given their roles in the controlling and adjusting of movements and in adaption to environmental changes [57]. Unfortunately, nerve roots can be damaged, thereby disrupting complex and highly specialized neural networks, impairing neural signal transmission.

**Fig. 1:**
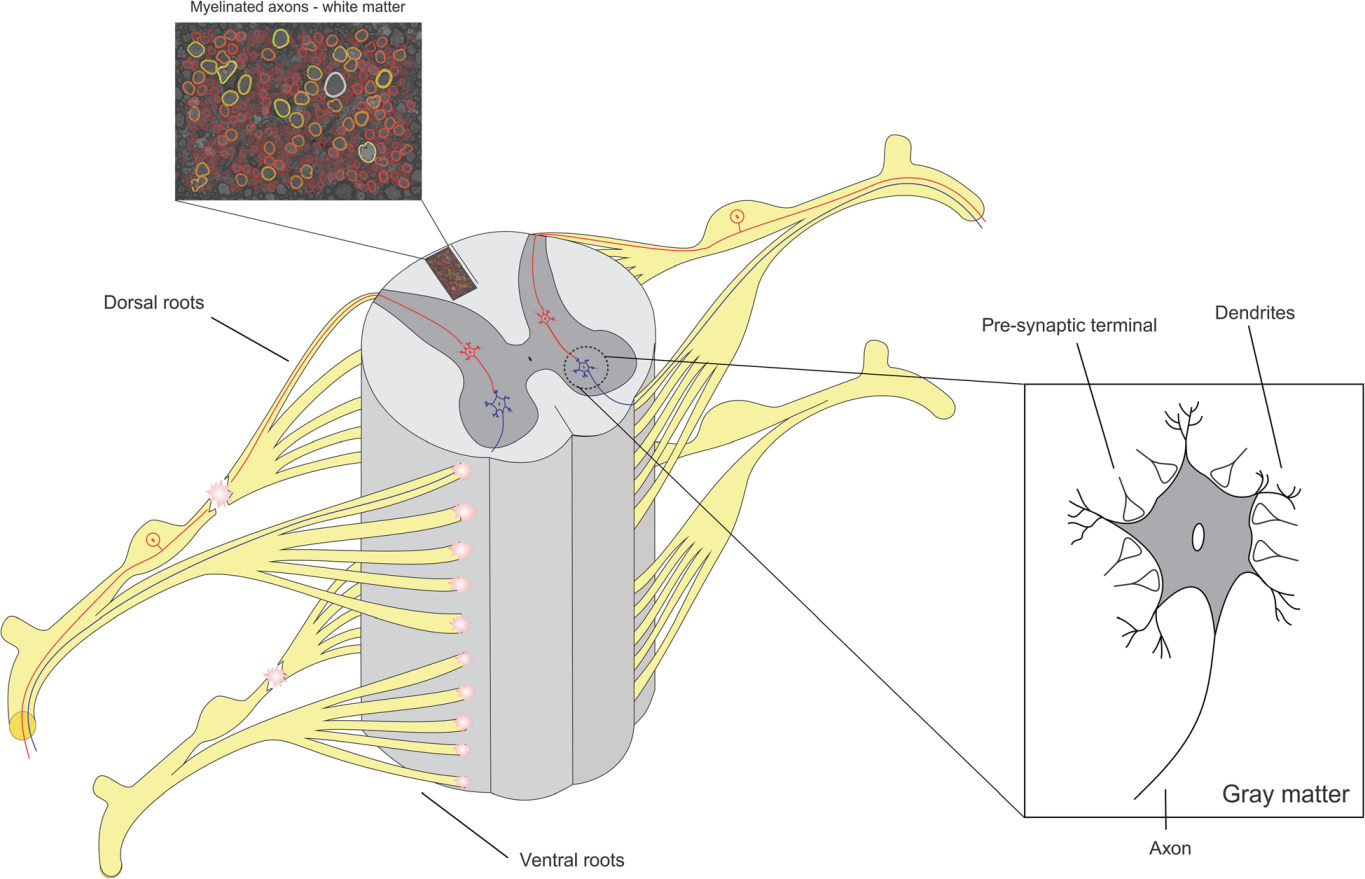
Schematic view of dorsal and ventral nerve roots. Sensory neurons bring sensory inputs through the dorsal roots, which are transmitted to the motoneurons via interneurons. Motoneurons send their axons through ventral roots that innervate target muscles. Dorsal root lesion and ventral root avulsion have been used to evaluate the efficacy of the CEVAP heterologous fibrin sealant (HFS) for CNS repair and regeneration. The inset shows presynaptic terminals in apposition to motoneurons that retract after injury (synapse pruning). Myelinated axons located in the white matter are highlighted (*top left*) by using AxonSeg, an open source software for axon morphometry [58]

A schematic view of dorsal and ventral nerve roots, as well as structures of gray and white matter, are represented in Fig. 1. It also illustrates the ventral root avulsion and dorsal root section lesions. Axons in the white matter are highlighted with the program AxonSeg, available online [58].

Proximal root injury, differently from peripheral nerve lesion, results in extensive degeneration of adult motoneurons and loss of sensory feedback since axons cannot regenerate into the spinal cord [59, 60]. When this injury happens in an abrupt traction, it is called avulsion [61, 62]. Such lesion or damage frequently occurs in severe brachial plexus injuries due to the high impact of the trauma [61]. In cats and dogs, avulsion is normally associated with being hit by vehicles [63, 64]. Nevertheless, in humans, it often happens in vehicle or sport accidents with limb traction or shoulder depression. In such accidents, the brachial plexus can be damaged when the head is pushed away from the shoulder [57, 65]. Compression or crushing, industrial trauma, and iatrogenic injury are mechanisms that can also produce root avulsion [61, 62]; and a similar lesion can also happen in the newborn during childbirth [57].

The disconnection of spinal motoneurons from the muscle fibers interrupts the anterograde flow of neurotrophic factors, leading to neuronal degeneration and skeletal muscle paralysis. On the contrary, deafferentation after dorsal root disconnection does not result in significant dorsal root ganglia neuronal death, although it leads to loss of sensory feedback [66, 67]. Overall, root lesions trigger several long-lasting structural changes in the CNS, affecting not only local spinal cord circuits but also the entire motor pathway, including the motor cortex [68, 69].

Extensive synaptic plasticity occurs in the motoneuron cell body in response to ventral root avulsion, with preferential loss of excitatory inputs [70, 71]. In turn, such imbalance of synaptic connections impairs voluntary movements and may cause neuropathic pain and/or hyperalgesia. Additionally, proximal lesions also break the blood–brain barrier, facilitating the influx of blood-borne cells, increasing inflammation and glial reaction [65, 72–76]. Reactive astrocytes participate in presynaptic terminal retraction from the surface of injured motoneurons, so that reducing inflammation usually preserves spinal cord circuits and facilitates regeneration [74, 77].

The clinical effect of root injury is not only restricted the loss of limb function ipsilateral to the lesion, but also several other subsequent clinical complications, such as meningeal cysts, intractable pain, impaired blood circulation, herniation and monoplegia [57, 62]. From the point of view of the patient, brachial plexus injury is devastating, leading to unemployment, economic hardship, and depression.

Poor recovery of function after brachial plexus avulsion can occur due to considerable distances through which motoneuron axons must regenerate to reconnect with the target muscles and the slow growth velocity of the regenerating axons, which cannot reach muscles before irreversible atrophy [78–80]. Also, when regenerating, afferent axons from the dorsal root ganglia reach the inhibitory environment of the spinal cord, being unable to reenter the CNS and reestablish functional connections [81]. On this matter, various attempts to promote regeneration after root lesion have been reported. Previous efforts to repair ventral roots were performed in rats by Carlstedt et al. [82] followed by Cullheim et al. in cats [83]. In these studies, the avulsed ventral roots were reimplanted on the surface of spinal cord lateral funiculus. A similar technique was applied to humans; however, with limited success [57, 84]. Further experimental ventral root implant approaches were carried out using 9/0 non-absorbable sutures (EthilonH®), lithium chloride, tissue glue (TisseelH®), fibrin sealant (TissueCol®; Baxter BVUtrecht, the Netherlands), nerve grafting, biodegradable scaffolds and nerve transfer [79, 85–91]. For dorsal root repair, some promising results towards regeneration were obtained by using inhibitors of chondroitin sulfate proteoglycans, myelin associated proteins, and by knocking down neurotrophin receptors [92–103].

The heterologous fibrin sealant derived from snake venom (HFS), alone or in association with cell therapy, has already shown promising results in the treatment of dorsal and ventral root injuries [67, 104]. Figure 1 shows the dorsal root rhizotomy. The HFS usage to reconnect ventral and dorsal roots also resulted in the statistically significant preservation of injured motoneurons, improved synaptic circuitry recovery, upregulation of trophic factors, and substantial recovery of sensory and motor function [67, 104–107]. Such studies provide a novel approach for treating spinal cord root lesions, aiming at restoring CNS/PNS interface integrity.

**Fig. 2:**
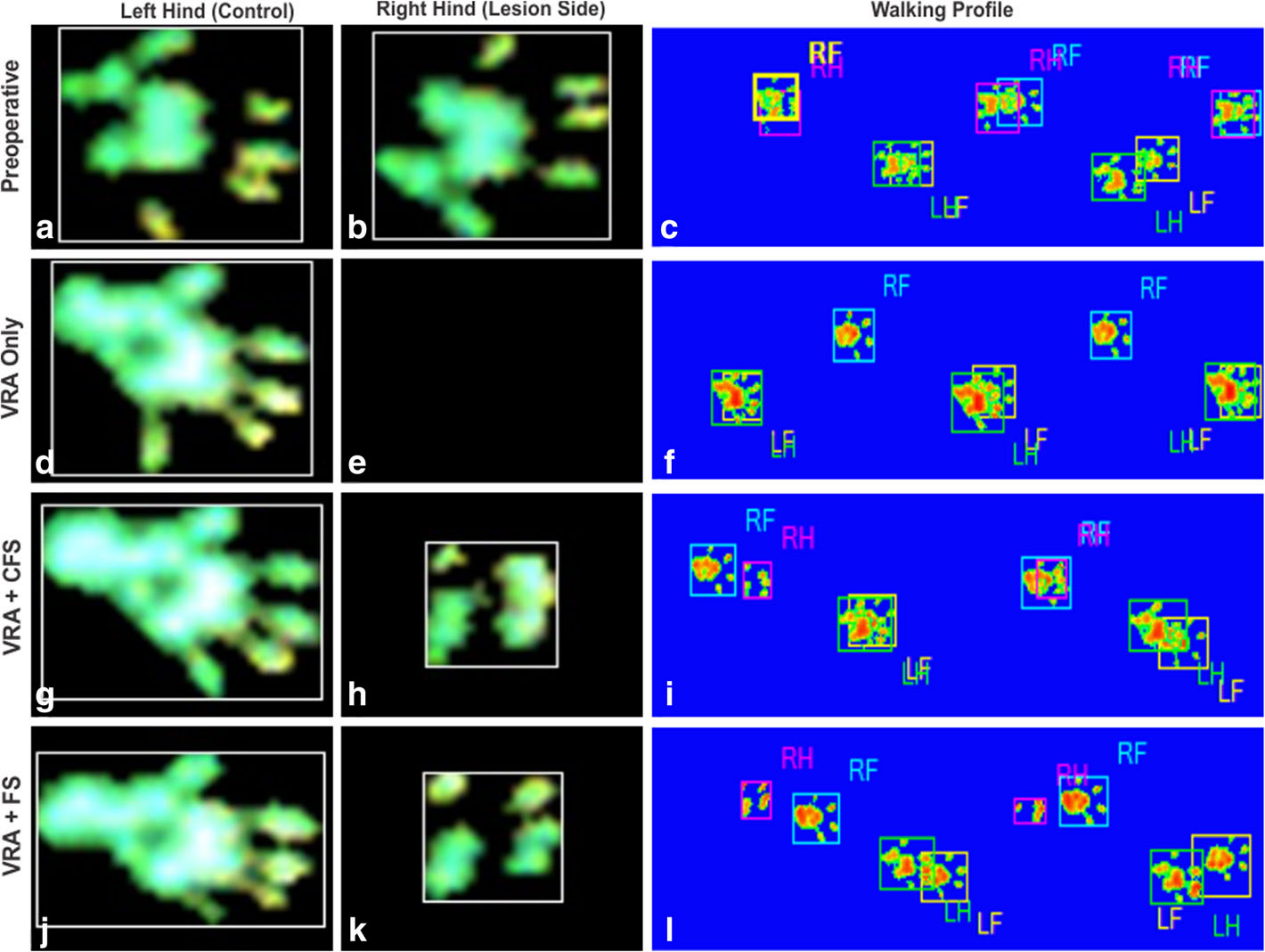
Paw prints and walking profile after ventral root avulsion and treatment with two different fibrin sealants, obtained with the CatWalk System (Noldus®). **a**-**c** Preoperative; **d**-**f** ventral root avulsion (VRA) only; **g**-**i** VRA followed by reimplantation with new heterologous fibrin sealant derived from snake venom (VRA + HFS); **j**-**l** VRA followed by reimplantation with commercial fibrin sealant (VRA + FS). It is possible to observe that (**h** and **k**) root reimplantation results in paw print partial recovery, whereas (**e**) avulsion alone leads to permanent paralysis

Vidigal de Castro et al. [107] showed a significant restoration of weight-bearing capacity following ventral root avulsion (VRA) and reimplantation with the heterologous (HFS) and commercial fibrin sealant (FS), showed by the overview of CatWalk System (Fig. 2) and Additional file 1 (VRA only), Additional file 2 (VRA + HFS) and Additional file 3 (VRA + FS).

## Peripheral nervous system

Peripheral nerve injuries lead to the disconnection of the nervous system with target organs, resulting in paralysis and numbness. Incomplete injuries usually cause pharmacologically resistant neuropathic pain [108]. Thus, the primary concern after nerve lesion is to secure the anatomical continuity, allowing regeneration of the axons towards the periphery.

End-to-end coaptation, with or without grafting is the gold-standard technique used to repair a sectioned peripheral nerve [109, 110]. Thus, the surgical approach depends on the degree of the lesion. Direct nerve repair with epineural suturing is possible when a tension-free adjustment and adequate vascularization can be achieved. When there is a gap between the stumps, generating significant tension for direct epineural repair, the interposition of autologous nerve grafts is required. In acute and clean nerve transection, the primary repair should be performed as soon as possible to improve neuronal survival and decrease fibrosis of the distal stump.

Minimizing the number of sutures can also decrease iatrogenic nerve tissue trauma. In this sense, alternative repair techniques have been studied in order to improve the stability of end-to-end coaptation. Tissue adhesives, such as the fibrin sealant, can either supplement or replace sutures and present advantages including reduction of surgery time [111, 112]. Besides, the FS may reduce suture-associated inflammation and enhance axonal regeneration [113].

A study comparing the use of FS alone, suturing and the combination of both techniques after sciatic nerve injury showed that fibrin sealant presented better results than suturing considering recovery of evoked motor action potential [114]. Other studies comparing the use of FS associated with peroneal nerve tubulization demonstrate that FS allows nerve regeneration and functional recovery without formation of neuroma [115, 116].

End-to-side coaptation has been proposed to repair peripheral nerves in the absence of the proximal stump. When associated with FS in rats, a greater number of regenerating fibers and improved motor recovery were observed [117].

Additional to FS coaptation, the use of neuroprotective and pro-regenerative substances, such as atorvastatin, was analyzed after sciatic nerve lesion demonstrating beneficial effects on muscle strength [118–121].

Wood et al. [122] evaluated the effects of glial cell-derived neurotrophic factor (GDNF) microspheres associated with FS, showing improvement in axonal regeneration and size of regrown axons. Tubular conduits prepared from FS can also improve short- and long-term regeneration following peripheral nerve injury, with regard to axonal sprouting and muscle weight recovery [123, 124]. Also, the evaluation of FS with bone marrow mononuclear stem cells on sciatic nerve injury demonstrated better results compared with FS alone as to morphometric parameters [125].

Importantly, the better understanding of nerve regeneration approaches requires careful evaluation of motor and sensory behavior. Such functional recovery is crucial for validation of morphological and molecular (e.g. gene expression) data [126]. In this sense, our group has dedicated much effort to combine reparative approaches with histological and molecular analyses and behavioral tests in order to improve the completeness of the results and findings [127, 128].

The fibrin sealant derived from snake venom (HFS) has been used for rat neonatal sciatic nerve coaptation facilitating the regenerative process. Furthermore, the comparison between HFS with another commercially available sealant (FS) revealed that both present similar performance in peripheral nerve repair [127]. Additional files 4, 5 and 6 illustrate normal gait pattern, evaluation after neonatal sciatic nerve axotomy and following coaptation, respectively.

An early study comparing HFS with other commercially available sealants showed that the new sealant promoted adequate sciatic nerve adherence and repair, highlighting that the nerve without repair showed extensive fibrosis and absence of nerve fibers [129]. More recently, another study using HFS, performed to evaluate functional recovery following sciatic nerve coaptation, showed improved recovery of neurophysiological parameters relative to action potential and muscle reinnervation [130]. The use of low-level laser therapy (LLLT) was also tested with HFS to observe the collateral repair of axons originating from the vagus nerve to the interior of a sural nerve graft, demonstrating that the HFS supports axonal regeneration [131]. Cartarozzi et al. [128] also observed sciatic nerve regeneration after combining mesenchymal stem cells and HFS in a polycaprolactone-based tubular prosthesis after nerve transection. CEVAP heterologous fibrin sealant scaffold combined with cell therapy improved Schwann cell reactivity, myelination and gait recovery.

## Conclusions

Biological sealants have long been used in research to provide a scaffold for substances and regrowth of axons and have been employed in neurosurgery for over 20 years without inducing damage to the nervous system [86, 101, 102, 132]. Sealant efficacy is similar or even better when compared to sutures in most of the cases [133–137]. However, commercial sealants have the disadvantage of using human blood that can lead to eventual transmission of infectious diseases, necrosis, and seroma formation [2].

As to the repair of nerves, the ideal sealant must possess specific biological, mechanical and structural properties, while presenting minimal risk of disease transmission, antigenicity, and toxicity. Furthermore, the sealant should not induce fibrosis, that can lead to nerve compression, and should not act as a barrier to axon regeneration, thereby preserving normal axon architecture. Adherence produced by the sealant should provide adequate mechanical strength to avoid nerve rupture, providing a stable scaffold for axonal growth. Additionally, it should be easy to handle, reducing operative time.

Taking all the above into account, the new heterologous fibrin sealant from snake venom (HFS) represents a consistent alternative, since it is produced without human blood to avoid transmission of infectious diseases. Its formulation can be customized to surgical needs; the clotting time can be adjusted and degradation time can be controlled. Moreover, HFS prevents fluid loss, promotes tissue adhesion, reduces surgery time and decreases hemorrhage [2, 127]. In addition, it is cheaper than commercial heterologous sealants, since the technology and production processes have been optimized [2, 4].

## Additional files

Additional file 1: Video showing loss of weight-bearing capacity following ventral root avulsion (VRA), 12 weeks post-surgery, in the the CatWalk System. (AVI 10692 kb)
Additional file 2: Video showing restoration of weight-bearing capacity following ventral root avulsion (VRA) and reimplantation with the heterologous fibrin sealant (HFS), 12 weeks post-surgery, CatWalk System. (AVI 12966 kb)
Additional file 3: Video showing restoration of weight-bearing capacity following ventral root avulsion (VRA) and reimplantation with commercial fibrin sealant (FS), 12 weeks post-surgery, CatWalk System. (AVI 15923 kb)
Additional file 4: Video showing normal gait pattern evaluation in a control animal. (AVI 17628 kb)
Additional file 5: Video showing gait pattern evaluation after neonatal (P2) sciatic nerve axotomy followed by coaptation: sciatic nerve repair with the heterologous (HFS) sealant. (AVI 14347 kb)
Additional file 6: Video showing gait pattern evaluation after neonatal (P2) sciatic nerve axotomy followed by coaptation: recovery following nerve repair using commercial fibrin sealant (FS), 12 weeks post-surgery. (AVI 10675 kb)

## Abbreviations

BDNF: Brain-derived neurotrophic factor
CEVAP: Center for the Study of Venoms and Venomous Animals (Brazil)
CNS: Central nervous system
CSF: Cerebrospinal fluid
FGF: Fibroblast growth factor
FS: Fibrin sealant
GDNF: Glial cell-derived neurotrophic factor
HFS: CEVAP heterologous fibrin sealant
IL­10: Interleukin 10
IL­13: Interleukin 13
IL-1β: Interleukin 1-beta
IL­4: Interleukin 4
iPS: Inducible pluripotent stem cells
iPS-FS: Inducible pluripotent stem cells mixed with fibrin sealant
LLLT: Low-level laser therapy
MCAO: Middle cerebral artery occlusion
NGF: Nerve growth factor
PEG: Polyethylene glycol
PNS: Peripheral nervous system
SCI: Spinal cord injury
SFC: Subdural fluid collection
TNF: Tumor necrosis factor
VRA: Ventral root avulsion
